# Vitamin E for the Prevention of Contrast-Induced Nephropathy: A Systematic Review and Meta-Analysis

**DOI:** 10.7759/cureus.63256

**Published:** 2024-06-26

**Authors:** Ahmed Ali Awaji, Basel H Bakhamees, Nouf K Alalshaikh, Nawaf M Albelwi, Mead M AL-Zahrani, Khalaf F Alshammari, Shaden D Almutairi, Ilaf M Siraj, Taif N Aljaber, Raghad S Alnajdi, Shatha S Al-Majnooni, Abdulaziz Saeed Alserhani

**Affiliations:** 1 Arthroplasty and Lower Extremity Reconstruction Surgery, King Salman Armed Forces Hospital, Tabuk, SAU; 2 Faculty of Medicine, King Abdulaziz University, Jeddah, SAU; 3 Medicine, King Saud Bin Abdulaziz University for Health Sciences College of Medicine, Jeddah, SAU; 4 Internal Medicine, King Salman Armed Forces Hospital, Tabuk, SAU; 5 Public Health, Albaha University, Albaha, SAU; 6 Internal Medicine, King Salman Specialist Hospital, Hail, SAU; 7 Medicine, Ibn Sina National College for Medical Studies, Jeddah, SAU; 8 Medicine, Unaizah College of Medicine and Medical Sciences, Qassim University, Qassim, SAU; 9 Faculty of Medicine, Imam Mohammed Ibn Saud Islamic University, Riyadh, SAU; 10 Pharmacy, Umm Al-Qura University, Makkah, SAU; 11 Faculty of Medicine, King Khalid University Hospital, Abha, SAU

**Keywords:** vitamin e, tocopherol, meta-analysis, contrast induced nephropathy, clinical trials, acute kidney injury

## Abstract

Contrast-induced nephropathy (CIN) is a serious condition that may develop in patients undergoing diagnostic radiologic procedures. Several treatments have been assessed to prevent CIN development. This study aims to assess the efficacy and safety of vitamin E in the prevention of CIN compared to intravenous (IV) saline hydration. The literature search included MEDLINE/PubMed, Cochrane Central Register of Controlled Trials, the Web of Science, ProQuest, and Scopus for articles published until May 11, 2024, without language or time limits. The outcomes included the incidence of CIN, new-onset dialysis, and death (primary), as well as the change in serum creatinine and glomerular filtration rate (GFR) (secondary). Numerical and dichotomous outcomes were presented as standardized mean difference (SMD) and risk ratio (RR), respectively, with 95% confidence intervals (CI). Six clinical trials were included. Vitamin E was administered orally in varying doses, but one study used IV infusion. Vitamin E decreased the risk of developing CIN by 59% (n=5; pooled RR: 0.41; 95% CI: 0.25, 0.65; P<0.001) compared to IV hydration. None of the patients required renal replacement therapy. One patient on vitamin E died due to the occurrence of acute coronary syndrome. Vitamin E is a promising effective prophylaxis against CIN. However, the number of included studies and their sample sizes are small. The studies showed several limitations. There is a need for further high-quality clinical trials to ascertain the effectiveness of vitamin E compared to IV hydration and to compare vitamin E to other therapies, such as N-acetyl cysteine.

## Introduction and background

Contrast-induced nephropathy (CIN) is an iatrogenic acute kidney injury (AKI) that develops in some individuals following the intravascular administration of a contrast medium, with no other potential causes present [1]. The condition ranks as the third most common cause of AKI in hospitalized patients [1]. The incidence of CIN ranges from 2% to 30%, rising to between 20% and 30% in high-risk groups [2, 3]. The development of CIN is associated with prolonged hospitalization, increased healthcare costs, and a higher risk of mortality [4, 5], warranting effective protective measures to reduce its incidence.

Typically, CIN is diagnosed when serum creatinine concentrations show either an absolute increase of ≥ 0.5 mg/dL or a relative increase of ≥ 25% within 48 to 72 hours following the administration of an intravascular contrast medium. Increases in serum creatinine may peak up to three to five days after exposure to the contrast medium [6].

Patients at increased risk for developing CIN include those above the age of 75 years, female patients, and individuals with previous kidney insufficiency, diabetes mellitus, multiple myeloma, hypertension, congestive heart failure, or those receiving nephrotoxic drugs such as non-steroidal anti-inflammatory drugs, amphotericin B, aminoglycosides, and angiotensin-converting enzyme inhibitors [7].

The pathophysiology of CIN involves the interaction of several complex mechanisms. Contrast agents can induce renal vasoconstriction, resulting in medullary ischemia. The resultant hypoperfusion leads to the generation of reactive oxygen species, which cause additional renal injury [8]. Furthermore, contrast agents exert a direct toxic effect on renal tubules [9-12]. Oxidative stress appears to play a pivotal role in the development of CIN [13].

The identification of prophylactic methods against CIN has been the focus of numerous studies, both those investigating animal models and clinical trials. The tested hypotheses of prophylactic measures were based on targeting the mechanisms involved in causing CIN. Volume expansion by the administration of isotonic saline infusion increases tubular urine flow, decreases the concentration of the contrast agent, reduces the secretion of vasoconstrictors, and diminishes the release of reactive oxygen species [9]. Other tested methods include vasodilation using phosphodiesterase 5 inhibitors (such as fenoldopam and dopamine) to decrease renal vascular resistance [14-16], as well as the prevention of oxidative stress. The latter method tested several antioxidant agents such as N-acetylcysteine [16-18], vitamin C [17, 19], and vitamin E [20-23]. According to the guidelines issued by Kidney Disease: Improving Global Outcomes (KDIGO), IV hydration and N-acetylcysteine are routinely used to guard against CIN [24].

Although several clinical trials and systematic reviews assessed the role of other antioxidants added to IV hydration in preventing CIN, the results were inconsistent across the studies [25-27].

Vitamin E refers to a group of eight lipid-soluble antioxidant compounds synthesized in plants: 4 tocopherols (α-, β-, γ-, δ-tocopherol) and 4 tocotrienols (α-, β-, γ-, δ-tocotrienol). Among these compounds, α-tocopherol exhibits the highest biological activity [28]. The present systematic review and meta-analysis aimed to assess the efficacy and safety of vitamin E in the prevention of CIN compared to intravenous (IV) saline hydration.

## Review

Methodology

The study was conducted according to the principles of the Cochrane Handbook for Systematic Reviews of Interventions, version 6, and reported following the Preferred Reporting Items for Systematic Reviews and Meta-Analyses (PRISMA) guidelines [29]. We included only randomized and non-randomized clinical trials published in English, without setting limitations on the publication time. Studies included patients exposed to an intravascular contrast medium and directly compared the administration of prophylactic vitamin E plus IV hydration to IV hydration alone. We excluded conference abstracts, duplicate reports, case reports, observational studies, review articles, editorials, clinical guidelines, and single-arm studies.

An online search was conducted using five databases: MEDLINE/PubMed, Cochrane Central Register of Controlled Trials (CENTRAL), Web of Science, ProQuest dissertation and theses, and Scopus. We did not use any search filters, and the search encompassed the time period from inception until May 11, 2024. The terms used for searching included "Contrast Media" AND ("nephropathy" OR "CIN") OR ("Acute Kidney Injury")) AND (("vitamin e" OR "vitamin E" OR "tocopherol" OR "Tocopherol" OR "Tocopherols")). The Polyglot Search Translator [30] from Systematic Review Accelerator (SRA), Bond University, was used in formulating the search terms.

The processes of online search, screening the titles and abstracts, and revising the full text of relevant articles were carried out by two independent reviewers. Any disagreements were resolved by consensus. We extracted the following data: (a) the study design, time span, inclusion and exclusion criteria, sample size, the used contrast medium, the diagnostic procedure, the dosage and regimen of vitamin E, the regimen of IV saline hydration, and the definition of CIN used by the study; (b) patients’ age, sex, baseline serum creatinine, and estimated glomerular filtration rate (eGFR); and (c) the outcomes: number of patients who were diagnosed with CIN, required dialysis, or died, as well as the change from baseline in serum creatinine and eGFR.

The study's measured outcomes are categorized into primary and secondary outcomes. The primary outcomes include the incidence of CIN, new-onset dialysis, and death. The secondary outcomes focus on the changes in serum creatinine levels and eGFR, providing a comprehensive overview of renal function and potential damage following contrast exposure. The assessment of the risk of bias (ROB) was performed using the ROB2 tool for randomized clinical trials (RCTs) [31], as all included studies were found to be RCTs. The ROB2 tool comprises five domains: randomization, deviations from the assigned treatment, missing data, measurement of the outcome, and selective reporting of the outcomes and results. Moreover, the overall ROB is assessed by selecting the highest level of ROB out of the five domains.

**Figure 1:**
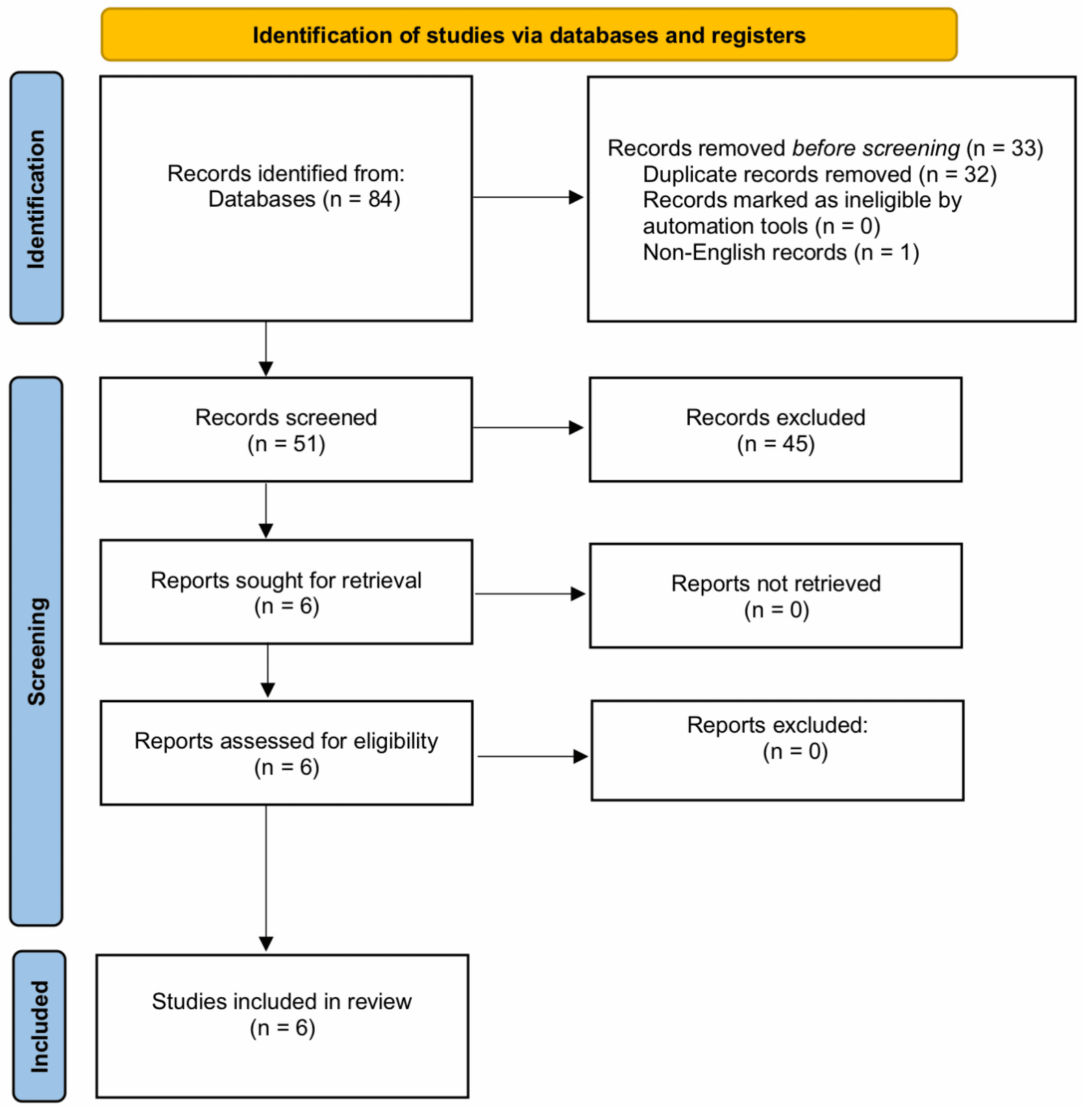
PRISMA flow chart for the literature search and study selection PRISMA: Preferred Reporting Items for Systematic Reviews and Meta-Analyses.

The meta-analysis was performed using the R Statistical language (version 4.4.0, R Foundation for Statistical Computing, Vienna, Austria) [32], using the packages meta (version 7.0.0, CRAN, Vienna, Austria) [33] and dmetar (version 0.1.0, CRAN, Vienna, Austria) [34]. We compared numerical outcomes and presented the difference using the standardized mean difference (SMD). For dichotomous outcomes, the risk ratio (RR) was calculated. We tested heterogeneity and identified significant heterogeneity if the p-value from the Cochran Chi-square test was <0.1 and/or the I² index was ≥50%. If no significant heterogeneity was detected, we pooled the studies’ findings using a fixed-effect model [35]. A p-value <0.05 was used to define statistical significance in the comparisons between the two groups. We did not carry out testing for publication bias as the number of eligible studies was less than 10. The search strategy yielded 84 records, out of which 32 were duplicates and removed while one record was not available in English. The remaining 51 records underwent screening of their titles and abstracts, and 45 records were excluded. The full texts of the remaining six records were obtained (20-23, 36, 37), and all six were eligible for inclusion in the present systematic review and meta-analysis (Figure 1).

All the included studies were randomized, parallel-group, controlled trials. Three studies were conducted in Iran [23, 36, 37], two studies in Thailand [20, 22], and one study in Austria [21] (Table 1).

**Table 1:** Characteristics of the included studies (n = 6) ACEIs: angiotensin-converting enzyme inhibitors; AKI: acute kidney injury; ARBs: angiotensin receptor blockers; ARF: acute renal failure; CA: coronary angiography; CHF: congestive heart failure; Cr: creatinine; ESRD: end-stage renal disease; NAC: N-acetyl cysteine; NR: not recorded; NSTE-ACS: non–ST-segment elevation acute coronary syndrome; RCT: a randomized controlled trial; STE: ST-segment elevation.

Study	Location	Time span	Inclusion criteria	Exclusion criteria
Tasanarong et al. [20]	Thailand	Jan 2006 to Jun 2007	Serum Cr ≥ 1.2 mg/dL and Cr clearance ≤ 60 mL/min (during 2 months before protocol)	ARF; ESRD on dialysis; unstable renal function (change in serum Cr > 0.5 mg/dL or > 25% within 14 days before study); allergy to contrast agents; mechanical ventilation; CHF; cardiogenic shock or emergent CA; receiving NAC, mannitol, diuretics, theophylline, dopamine, or contrast agents within 14 days before study; daily alpha-tocopherol in the week before study
Kitzler et al. [21]	Austria	Aug 2002 to Jul 2003	Age ≥18 years; serum Cr >1.25 mg/dL for males and 1.09 mg/dL for females; Not under renal replacement therapy	AKI; serum Cr increase in the enrollment period >0.2 mg/dL; receiving any antioxidant therapy within 4 weeks before the study; participation in a trial within 1 month before this study; allergy to the investigational drugs; current use of theophylline, dopamine, furosemide, or mannitol
Tasanarong et al. [22]	Thailand	Jan 2008 to Dec 2010	CKD (baseline eGFR ≤ 60 mL/min); undergoing elective CA and/or intervention	AKI, CKD Stage 5; unstable renal function (change in serum Cr ≥ 0.5 mg/dL, or ≥ 25%, within 14 days before study); allergy to contrast agents; mechanical ventilation; CHF, cardiogenic shock, or emergent CA; receiving NAC, mannitol, diuretics, theophylline, dopamine, ascorbic acid, or contrast agents within 14 days before study; daily α- or γ-tocopherol in the week before study
Rezaei et al. [23]	Iran	Feb 2014 to Jun 2015	Age ≥18 years; eGFR <60 mL/min/1.73 m^2^; undergoing CA; stable angina with ischemia or NSTE-ACS requiring an early invasive strategy	Acute STE myocardial infarction; high-risk NSTE-ACS warranting emergency CA (<2 hours); cardiogenic shock, pulmonary edema, overt heart failure and/or ejection fraction < 30%; ACS undergoing CA or angioplasty during the previous 5 days; sensitivity to contrast medium; recent administration of contrast medium; AKI; history of dialysis; pregnancy; newly prescribed ACEIs or ARBs; bleeding and/or coagulopathy diseases; receiving nephrotoxic drugs, vitamin E, vitamin C, or NAC within 48 hours before intervention
Samadi et al. [36]	Iran	NR	CKD patients with GFR < 60mL/min for at least 3 months	CKD stage 5; allergy to the contrast; mechanical ventilation; CHF; cardiogenic shock; urgent CA; receiving mannitol, theophylline, dopamine, ascorbic acid, or contrast media within 14 days before CA; taking alpha-tocopherol daily during the week before the study
Pishgahi et al. [37]	Iran	Jun 2019 to May 2020	DM types I and II; stable angina risk of acute myocardial infarction or ACS for diagnostic CA	Sensitivity to contrast mediums; cardiogenic shock; acute myocardial infarction; pulmonary edema; ARF; pregnancy; hemodialysis; ACS; underwent CA or angioplasty or injected any other contrast medium in the past 5 days; CKD with GRF <30 mL/min

All studies included patients undergoing elective diagnostic coronary angiography, except one study, which included patients referred for contrast computerized tomography [21]. The contrast agent used was Iopromide in three studies [20-22] and Iodixanol in one study [23], while two studies did not report the type of contrast agent [36, 37]. Vitamin E was administered orally in all studies except the study by Kitzler et al. [21]. The dosage of vitamin E varied widely across the studies. All patients received IV saline hydration before and after the contrast radiography. Saline was isotonic in all studies except that by Kitzler et al. [21], where saline 0.45% infusion was used. The definition of CIN was nearly similar across studies, but the time for measuring renal function differed, being 48 hours in most studies [20-22, 37]. The study by Rezaei et al. [23] assessed CIN within 72 hours after coronary angiography (Table 2).

**Table 2:** Diagnostic procedure and used definitions in the included studies (n = 6) CA: coronary angiography; SCr: serum creatinine.

Study	Procedure	Contrast medium	Vitamin E route and dosage	IV hydration	CIN definition
Tasanarong et al. [20]	Elective CA and/or intervention	Iopromide	Oral; 525 IU; at 48 hours, 24 hours, and in the morning before CA	Isotonic saline, 1 mL/kg/hr for 12 hours before and 12 hours after CA	The absolute increase in SCr > 0.5 mg/dL or a relative increase >25% in SCr at 48 hours after the procedure, compared to baseline measurements
Kitzler et al. [21]	CT	Iopromide	IV; 540 mg; at 12 and 6 hours before and then 6 and 12 hours after CT	Saline 0.45%, 1 mL/kg/hr for 12 hours before and after CT	Increase in SCr >25 % over the baseline value in the following 48 hours after CT
Tasanarong et al. [22]	Elective CA and/or intervention	Iopromide	Oral; 350 mg/day; initiated 5 days before the procedure and continued for 2 days after CA	Isotonic saline, 1 mL/kg/hr for 12 hours before and 12 hours after CA	The absolute increase in SCr > 0.5 mg/dL or a relative increase > 25% in SCr at 48 hours after the procedure, compared to baseline measurements
Rezaei et al. [23]	Elective CA	Iodixanol	Oral; 600 mg; at 12 hours before then 400 mg 2 hours before CA	Isotonic saline, 1 mL/kg/hr for 12 hours before and 12 hours after CA	Absolute increase ≥0.5 mg/dL or a relative increase ≥25% over baseline SCr concentration within 72 hours after administration of contrast media
Samadi et al. [36]	CA	NR	600 IU once daily; from the day before till 2 days after CA for a total of 4 days	Isotonic saline, 1 mL/kg/hr for 12 hours before and 12 hours after CA	NR
Pishgahi et al. [37]	CA	NR	Oral; 600 mg; 2 hours before and 40 mg 4 hours after CA	Isotonic saline, 1 mL/kg/hr for 12 hours before and 12 hours after CA	>0.5 mg/dL or 25% increase in SCr from baseline level within 48 hours

The baseline characteristics of the participants are summarized in Table 3.

**Table 3:** Summary of baseline criteria in the included studies (n = 6) eGFR: estimated glomerular filtration rate; F: female; M: male; IQR: interquartile range; N: number; NR: not recorded; SCr: serum creatinine; SD: standard deviation.

Study	Arms	N	Age (years), mean ± SD	Gender (M:F)	Baseline SCr (mg/dL), mean ± SD	Baseline eGFR (mL/min/1.73 m^2^), mean ± SD
Tasanarong et al. [20]	Vitamin E	51	68 ± 9	40:11	1.62 ± 0.44	NR
	Control	52	65 ± 11	36:16	1.67 ± 0.53	NR
Kitzler et al. [21]	Vitamin E	10	73.3 ± 11.9	6:4	1.37 ± 0.2	49.6 ± 11.4
	Control	10	74 ± 8.5	5:5	1.33 ± 0.12	48.2 ± 7.4
Tasanarong et al. [22]	Vitamin E	102	67 ± 9	80:22	1.58 ± 0.48	45 ± 13
	Control	101	66 ± 10	71:30	1.63 ± 0.53	43 ± 13
Rezaei et al. [23]	Vitamin E	149	66 ± 11	68:81	1.3 (IQR: 1.2–1.5)	45 (IQR: 39–53)
	Control	149	67 ± 10	69:80	1.3 (IQR: 1.2–1.5)	44 (IQR: 37–51)
Samadi et al. [36]	Vitamin E	63	≤ 75 years: 51 >75 years: 12	21:42	1.32 ± 0.46	1.21 ± 0.18
	Control	72	≤ 75 years: 63 >75 years: 6	33:39	1.31 ± 0.33	51.19 ± 7.55
Pishgahi et al. [37]	Vitamin E	94	65 ± 9.3	66:28	1.38 ± 0.22	NR
	Control	113	64 ± 7.4	78:35	1.31 ± 0.16	NR

The ROB was assessed using the ROB2 tool for all included trials. The summary of the ROB in each domain as well as the overall risk is provided in Table 4. 

**Table 4:** The risk of bias assessment for the included trials based on the ROB2 tool (n = 6)

Study	Randomization process	Deviations from intended interventions	Missing outcome data	Measurement of the outcome	Selection of the reported result	Overall
Tasanarong et al. [20]	Some concerns	Low	Low	Low	Low	Some concerns
Kitzler et al. [21]	Low	Low	Low	Low	Some concerns	Some concerns
Tasanarong et al. [22]	Some concerns	Some concerns	Low	Low	Some concerns	Some concerns
Rezaei et al. [23]	Low	Low	Low	Low	Low	Low
Samadi et al. [36]	High	High	Low	High	Some concerns	High
Pishgahi et al. [37]	Low	Low	Low	Low	Low	Low

The ROB regarding the process of randomization was low in three studies only [21, 23, 37], while two studies showed some concerns due to a lack of details about the generation of the random allocation sequence [20, 22] and unclear allocation concealment [22]. In addition, one study showed a high risk [36] of selection bias due to a lack of details about randomization and concealment as well as a lower prevalence of diabetes mellitus in the vitamin E group. Deviations from intended interventions were a concern in one study [22] as a per-protocol analysis of outcomes was done. Another study [36] presented a high risk due to unclear blinding of participants and carers, as well as the non-use of intention-to-treat analysis. The risk of missed outcome data was low in all studies. The measurement of outcomes showed low ROB except in one study where the risk was high [36] because the study did not define how CIN would be diagnosed. The risk of selective reporting of outcomes was low in three studies [20, 23, 37] but raised some concerns in the other three studies [21, 22, 36] due to the non-availability of a protocol that was finalized before conducting the study to compare the methodology.

Results of meta-analysis

Contrast-Induced Nephropathy

**Figure 2:**
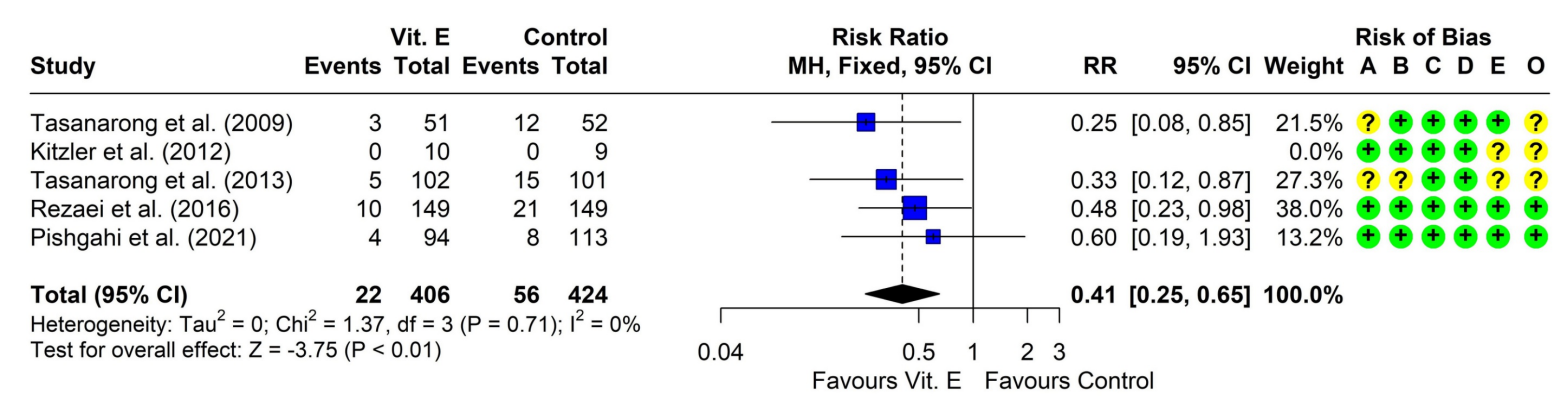
Forest plot showing pooling of the studies’ findings regarding the incidence of CIN CI: confidence interval; MH: Mantel-Haenszel method; RR: risk ratio; A: randomization process; B: deviations from intended interventions; C: missing outcome data; D: measurement of the outcome; E: selection of the reported result; O: overall risk of bias. Reference: [20-23, 37].

Five studies reported the incidence of CIN in the two groups [20-23, 37]. One study reported that none of the patients developed CIN in either group [21], so the study could not be included in the meta-analysis. The other four studies reported that the risk was reduced in the vitamin E group compared to those receiving only IV hydration. Heterogeneity testing was non-significant (chi² = 1.37, P = 0.713, I² = 0%), so the fixed-effect model was used. The pooled RR (95% CI) was 0.41 (0.25, 0.65), P < 0.001 (Figure 2). Leave-one-out analysis did not suggest the presence of influencing studies.

New-Onset Dialysis

Four studies [20-23] reported that none of the patients developing CIN required dialysis (Table 5).

**Table 5:** Summary of the outcomes in the included studies (n = 6) CIN: contrast-induced nephropathy; eGFR: estimated glomerular filtration rate; F: female; M: male; IQR: interquartile range; N: number; NR: not recorded; SCr: serum creatinine; SD: standard deviation.

Study	Arms	N	EOS SCr (mg/dL)	EOS eGFR (mL/min/1.73 m^2^)	Change in SCr (mg/dL)	Change in eGFR (mL/min/1.73 m^2^)	CIN	New-onset dialysis	Adverse events	Death
Tasanarong et al. [20]	Vitamin E	51	1.64 ± 0.59	NR	0.02 ± 0.33	NR	3	0	2	0
	Control	52	1.90 ± 0.87	NR	0.23 ± 0.71	NR	12	0	2	0
Kitzler et al. [21]	Vitamin E	8	1.36 ± 0.22	50.9 ± 11.3	0.03 ± 0.2	−1.0 ± 8.4	0	0	NR	0
	Control	8	1.36 ± 0.11	45.6 ± 5.8	0.03 ± 0.1	−1.3 ± 4	0	0	NR	0
Tasanarong et al. [22]	Vitamin E	102	1.59 ± 0.61	47 ± 16	0.01 ± 0.38	2 ± 9	5	0	NR	0
	Control	101	1.77 ± 0.85	43 ± 17	0.14 ± 0.64	0 ± 10	15	0	NR	0
Rezaei et al. [23]	Vitamin E	149	1.3 (IQR: 1.1–1.4)	49 (IQR: 41–59)	NR	NR	10	0	0	1
	Control	149	1.3 (IQR: 1.1–1.5)	49 (IQR: 39–55)	NR	NR	21	0	0	0
Samadi et al. [36]	Vitamin E	63	NR	NR	NR	2.67 ± 3.177	NR	NR	NR	NR
	Control	72	NR	NR	NR	2.72 ± 3.277	NR	NR	NR	NR
Pishgahi et al. [37]	Vitamin E	94	1.32 ± 0.30	NR	-0.06 ± 0.27	NR	4	NR	NR	NR
	Control	113	1.39 ± 0.30	NR	0.08 ± 0.23	NR	8	NR	NR	NR

Change in Serum Creatinine From Baseline

**Figure 3:**
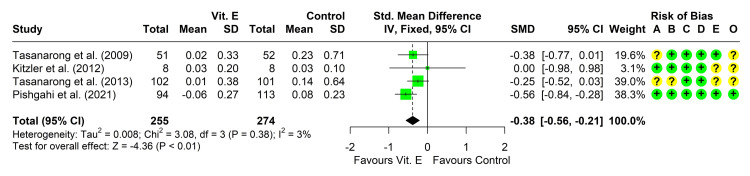
Forest plot showing pooling of the studies’ findings regarding the change from baseline in serum creatinine concentration CI: confidence interval; SMD: standardized mean difference; A: randomization process; B: deviations from intended interventions; C: missing outcome data; D: measurement of the outcome; E: selection of the reported result; O: overall risk of bias. Reference: [20-22, 37].

Five studies reported the change in serum creatinine levels from baseline in both groups [20-23, 37]. Four studies reported a lower change in serum creatinine in the vitamin E group compared to IV hydration [20, 22, 23, 37], with a significant difference in one study only [37]. The fourth study found comparable levels in the two arms [21]. Four studies reported enough data for the conduction of a meta-analysis [20-22, 37]. Heterogeneity testing was non-significant (chi² = 3.08, P = 0.380, I² = 3%), so the fixed-effect model was used to pool the results. The pooled SMD (95% CI) was -0.38 (-0.56, -0.21), P < 0.001 (Figure 3). Leave-one-out analysis suggested that the study by Pishgahi et al. may be influencing the results as its omission reduced the pooled SMD to -0.27 (-0.49, -0.06).

Change in eGFR From Baseline

**Figure 4:**
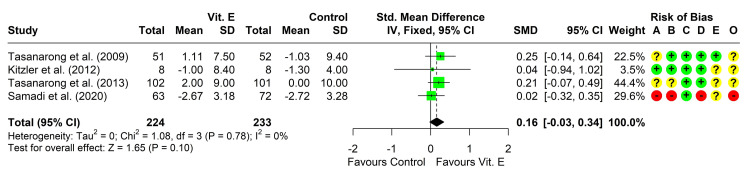
Forest plot showing pooling of the studies’ findings regarding the change from baseline in estimated glomerular filtration rate CI: confidence interval; SMD: standardized mean difference; A: randomization process; B: deviations from intended interventions; C: missing outcome data; D: measurement of the outcome; E: selection of the reported result; O: overall risk of bias. Reference: [20-22, 36].

Four studies reported the change in eGFR from baseline in both groups [20-22, 36], all of them reporting a non-significantly higher change in eGFR in the vitamin E group compared to IV hydration. Three studies reported enough data for the conduction of a meta-analysis [21, 22, 36]. Heterogeneity testing was non-significant (chi² = 1.08, P = 0.781, I² = 0%), so the fixed-effect model was used to pool the results. The pooled SMD (95% CI) was 0.16 (-0.03, 0.34), P = 0.098 (Figure 4). Leave-one-out analysis did not suggest the presence of influencing studies.

Death

Four studies [20-23] included death as one of the assessed outcomes. Only one patient died in the vitamin E group as reported by one study [23]. The cause of death was acute ST-segment elevation myocardial infarction (Table 5).

Adverse Effects

Only two studies assessed the development of intervention-related adverse effects [20, 23]. One study reported the occurrence of minor adverse effects in the form of nausea, vomiting, and abdominal discomfort on the first day, with a similar incidence in both groups (approximately 4%; Table 5). 

Discussion

CIN is a serious consequence that may develop in patients following exposure to an intravascular contrast medium during radiologic procedures [1]. The present meta-analysis aimed to assess the efficacy and safety of vitamin E in the prevention of CIN compared to IV saline hydration.

Based on the results of this meta-analysis, we found that vitamin E plus IV hydration reduced the incidence of CIN by 59% (RR (95% CI): 0.41 (0.25, 0.65), P < 0.001). Out of five studies, only one study reported that none of the patients developed CIN in either group [21]. This could be explained by the low dose of contrast medium (100 mL) that was used in that study.

The protective effect of vitamin E could be attributed to the decreased release of malondialdehyde and glutathione as markers of oxidative stress, which was elicited in rat models of inflammation [38, 39]. In addition, vitamin E may upregulate the expression of HSP70, SOD, and nitric oxide, which contribute to the elimination of free radicals, leading to decreased protein and lipid peroxidation in the renal cellular membrane and the amelioration of kidney injury, as reported in animal models of renal ischemia/reperfusion [40-42] and contrast-induced renal injury in rats [43].

The change in serum creatinine levels and eGFR showed favorable results in the direction of vitamin E intake; however, the results were not statistically significant. This may partially be explained by the small percentage of patients who developed CIN in either group, thus the change was not enough to reach statistical significance. The sample size included in the RCTs was also small, so the studies may not be powered enough to detect a significant difference in the change from baseline levels. The developed CIN was transient and not of a severe grade as none of the patients required dialysis. However, all the studies were short-term and did not report any findings beyond the 48 to 72 hours of observation after exposure to the contrast medium. Serum creatinine levels may still peak up to five days following exposure [6]. Previous studies on CIN reported that about 15-20% of patients suffered from persistent deterioration of kidney function [44, 45].

Previous meta-analyses reported nearly similar findings, though they included fewer studies than the current meta-analysis. A meta-analysis by Ali-Hasan-Al-Saegh et al. [46] included only two studies on vitamin E (20, 22) and concluded that vitamin E could significantly decrease the risk of CIN (OR (95% CI): 0.5 (0.27-0.92); p = 0.026), but could not significantly decrease the mean level of serum creatinine (SMD (95% CI): -0.25 (-0.46 to -0.05); p = 0.1).

Another meta-analysis by Cho et al. [47] included four studies [20-23]. They reported a higher reduction in the risk of CIN with vitamin E intake (RR (95% CI): 0.38 (0.22, 0.63); P < 0.010). In contrast to our results, they found that vitamin E reduced serum creatinine increase after exposure to contrast medium (SMD (95% CI): −0.27 (−0.49, −0.06); P = 0.010), while the difference in the changes of GFR did not reach statistical significance (SMD (95% CI): 0.21 (−0.01, 0.43); P = 0.060). Xu et al. [27] performed a meta-analysis based on these same four studies but investigated only the incidence of CIN, with a similar result (RR (95% CI): 0.39 (0.24, 0.62); P < 0.001) to that of Cho et al. [47]. However, they did not compare the changes in renal function tests between the two groups.

The most recent meta-analysis by Monami et al. [48] was also based on those four trials and reported similar findings regarding the reduction in the incidence of CIN (RR (95% CI): 0.34 (0.19, 0.59); P < 0.001), but with a non-significant difference regarding the changes from baseline in serum creatinine and eGFR. The present meta-analysis thus includes two more clinical trials than the previous meta-analyses.

The slight differences between the aforementioned analyses, despite depending on the same studies, are due to the use of different models to pool the results (fixed-effect and random effects models) and the difference in the effect size used (odds ratio or RR).

Overall Completeness, Applicability, and Quality of the Evidence

The evidence from the present meta-analysis suggests that vitamin E has a protective effect against CIN and can effectively reduce the risk of its development. However, the included studies showed some limitations, which call for further investigation before recommending the routine use of vitamin E in clinical practice. Most studies included a small sample size, and the incidence of CIN was relatively smaller than that reported in the literature in high-risk patients. Another concern is that the duration of observation ranged from 48 to 72 hours only after the procedure, so we cannot rule out that other undetected CIN cases developed after 72 hours.

If the effectiveness of vitamin E is confirmed, it can be an optimal protective agent as its pharmaceutical preparations are relatively inexpensive and its safety profile is good [49].

## Conclusions

The incidence of CIN was reduced with vitamin E administration, indicating a substantial protective effect of vitamin E. There was no significant heterogeneity among the included studies. In addition, the change in serum creatinine levels from baseline was significantly lower in the vitamin E group. The trials reported no need for new dialysis and minimal adverse effects, suggesting that vitamin E supplementation is a safe and effective intervention for reducing the risk of CIN in patients undergoing contrast radiography.

However, the number of included trials and their sample sizes are small, and the quality of some of the included trials had limitations, particularly regarding randomization and blinding procedures. In addition, the studies varied in the dosage and timing of vitamin E administration. Further research with robust designs and standardized protocols is needed to confirm these findings, determine the optimal dose and timing of vitamin E for the prevention of CIN, and compare vitamin E with other therapies, such as N-acetylcysteine.
